# Cav2.3 R-type calcium channels: from its discovery to pathogenic de novo CACNA1E variants: a historical perspective

**DOI:** 10.1007/s00424-020-02395-0

**Published:** 2020-06-11

**Authors:** T. Schneider, F. Neumaier, J. Hescheler, S. Alpdogan

**Affiliations:** Universitat zu Koln, 50931 Köln, Germany

**Keywords:** CACNA1E, Developmental and epileptic encephalopathy, Activation gate, Voltage sensor, Splice variants

## Abstract

So-called pharmacoresistant (R-type) voltage-gated Ca^2+^ channels are structurally only partially characterized. Most of them are encoded by the CACNA1E gene and are expressed as different Ca_v_2.3 splice variants (variant Ca_v_2.3a to Ca_v_2.3e or f) as the ion conducting subunit. So far, no inherited disease is known for the CACNA1E gene but recently spontaneous mutations leading to early death were identified, which will be brought into focus. In addition, a short historical overview may highlight the development to understand that upregulation during aging, easier activation by spontaneous mutations or lack of bioavailable inorganic cations (Zn^2+^ and Cu^2+^) may lead to similar pathologies caused by cellular overexcitation.

## Introduction

In the human gene of the pharmacoresistant Ca_v_2.3/R-type calcium channel, *de novo* pathogenic mutations were detected in a group of 30 individuals with developmental and epileptic encephalopathies [13]. The publication represents the first comprehensive investigation in humans with structural variations in this widely expressed voltage-gated calcium channel, together with two earlier reports, in which single cases were mentioned [4, 6].

**Fig. 1:**
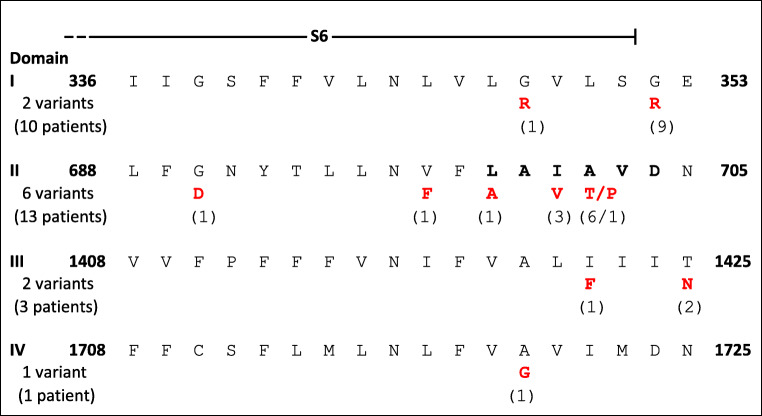
Alignment of the cytoplasmic parts from the Ca_v_2.3 S6 segments including 11 out of 14 identified disease-causing missense mutations, GenBank L27745.2 (inspired by Fig. 1 and Fig. S3 in Helbig et al., 2018). Note that for the mutants identified in domain II, recombinant studies have shown that the distal part of Ca_v_2.3 is important for the stability of the open state of Ca_v_2.3 [32]. Further, the first evidence for a strong electromechanical coupling between S4–S5 and the S6 of domain II came from a double mutant cycle analysis of the human Ca_v_2.3 confirming the hypothesis that leucine-596 in the IIS4–S5 linker couples strongly to the distal residues in IIS6 (**L A I A V D**) labelled in bold in this figure [40]. Three out of 30 mutations were located in IS5 (L228P), IIS4-5 (I603L) and IIIS6-IVS1 (G1430N)

Based on a short historical overview for the performed basic research, the path and the reasons for an improved understanding of the reported human mutations will be described. Interestingly, most of the channel mutations cluster within the cytoplasmic ends of the four S6 transmembrane segments (Fig. 1), which constitute part of the Ca_v_2.3-channel activation gate.

## “Activation gate” as a functional domain in voltage-gated ion channels

Ion channels represent transmembrane proteins, which are linked by their special structure and gating properties to many physiological functions, including cardiac and neuronal excitability. Ion channels can be either open or closed, and they contain structural elements, which are connected to the transition between these two states. The word “gate” is used to describe this concept, and “gating” is the process whereby the gate is opened and closed [1].

Ligand-gated and voltage-gated channels are activated by different processes but may both include the movement of some internal parts of the molecule to produce an effect in a different part of it to open the permanent pathway permitting the movement of ions. The cytoplasmic parts from the Ca_v_2.3 S6 segments (Fig. 1) represent at least the major “internal part” of the molecule, which causes activation of the channel to open it properly and precisely.

Much more is known by crystallography and mutational analyses about the structural details, which help to convert electrical signals generated by small ion currents across cell membranes to tune all rapid processes in biology and especially in voltage-gated Ca^2+^ and Na^+^ channels [8]. These channels contain a tetramer of membrane-bound domains including a positively charged S4 segment. Voltage-dependent activation drives the outward movements of these positive gating changes in the voltage sensor via a “sliding-helix mechanism”, which leads to a conformational change in the pore module that opens its intracellular activation gate (for further details related to the “chemical basis for electrical signalling”, see [8]).

Another recent review specializes on the Cav1.2/L-type Ca^2+^ channel, which is important for the plateau of the cardiac action potentials, muscle contractions, generation of pacemaker potentials, release of hormones and neurotransmitters, sensory functions and regulated gene expression [14]. Based on gating studies using biophysical and pharmacological studies as well as mathematical simulations, the role of voltage sensors in channel opening was analysed. The gating process is determined by distinct sub-processes, the movements of the voltage-sensing domains (the charged S4 segments) and the opening and closure of S6 gates (for further details related to the individual transitions during activation, see [14]).

The first structural details for the potential coupling between voltage sensors and the pore region came from the crystals of K^+^ channels [10]. The question arose: how the movement of the S4 segment is transmitted to the S6 helix to open the gate upon depolarization? In the electromechanical coupling model designed for the K_v_1.2 channel [20], the S4-S5 linker was located within atomic proximity (4–5 A) of the S6 helix. Thus, it may interact with the latter in the closed state of the channel and was confirmed by double mutant cycle analysis for the expressed human Ca_v_2.3 channel (for further details, see [40]).

## History – detection of R-type and “E-type” voltage-gated calcium channels (Tab. 1)

R-type (or initially called “E-type” [33]) Ca^2+^ channels were identified as the second last member of the group of high-voltage activated (HVA) Ca^2+^ channels [29]. They are divided into two subfamilies, (i) the L-type channels containing Ca_v_1.1-, Ca_v_1.2-, Ca_v_1.3- and Ca_v_1.4-α1 subunits as the ion-conducting pore and (ii) the non-L-type channels containing Ca_v_2.1(P-/Q-type)-, Ca_v_2.2(N-type)- and Ca_v_2.3(R-type)-α1 subunits as the proteins containing the ion-conducting pore. Low-voltage activated (LVA) Ca^2+^ channels were structurally defined later by *in silico cloning* [28, 30] and contain the α1 subunits of T-type channels (Ca_v_3.1-, Ca_v_3.2- and Ca_v_3.3--1) [42] with less homology to two former subfamilies Ca_v_1 and Ca_v_2. The ion-conducting subunit may be in most cases associated with a set of auxiliary subunits [9] and additional interaction partners as proteins binding to cytosolic loops or competing with auxiliary subunits [16, 17]. Within the native environment, they may typically function in the context of macromolecular signalling complexes including various upstream modulators and downstream effectors, which may be kept together by additional adapter and scaffold proteins [7].

**Table 1:** Time table for the structural and functional identification of Ca_v_2.3 variants in rabbit, ray, rat, mouse and human. A generalized and systematic overview for the Ca_v_2.3 splice variant nomenclature was published [27]

Year	Species	GenBank accession number	Nomenclatures (in bold are the present systematic names)	Miscellaneous	References
1992	Rabbit (*Oryctolagus cuniculus*)	X67855, X67856	BII-1, BII-2	Deduced primary sequence, no functional expression yet	[25]
1993	Ray (*Discopyge ommata*)	L12531	doe-1, R-type, **Ca**_**v**_**2.3**	Rapid inactivating channel	[12, 15, 43]
1993	Rat	L15453	rbe-II, R-type	Shorter amino terminus	[37]
1994	Human, foetal	L27745.2	E-type, α1Ed, **Ca**_**v**_**2.3d**	Longer splice variant, including exon 19 and exon 45 encoded insertions	[26, 33]
1994	Human, adult	L29384 L29385	α1E-1, **Ca**_**v**_**2.3a** α1E-3, **Ca**_**v**_**2.3c**	Lacking exon 19 and 45 Lacking exon 45 only	[41]
1994	Mouse	L29346	α1E-3, **Ca**_**v**_**2.3c**	Lacking exon 45 only	[41]
1994	Rabbit	X67856	BII-2, class E, **Ca**_**v**_**2.3**	Functional expression	[39]
1998	Rat, mouse, human	(PCR fragments)	α1Ee, **Ca**_**v**_**2.3e**	Predicted novel splice variant in cardiac and endocrine cell lines	[38]
2002	Rat	AY029412	α1E7, **Ca**_**v**_**2.3e**	Cardiac cell line, distribution in atrium and ventricle	[18, 21, 22]

**Fig. 2:**
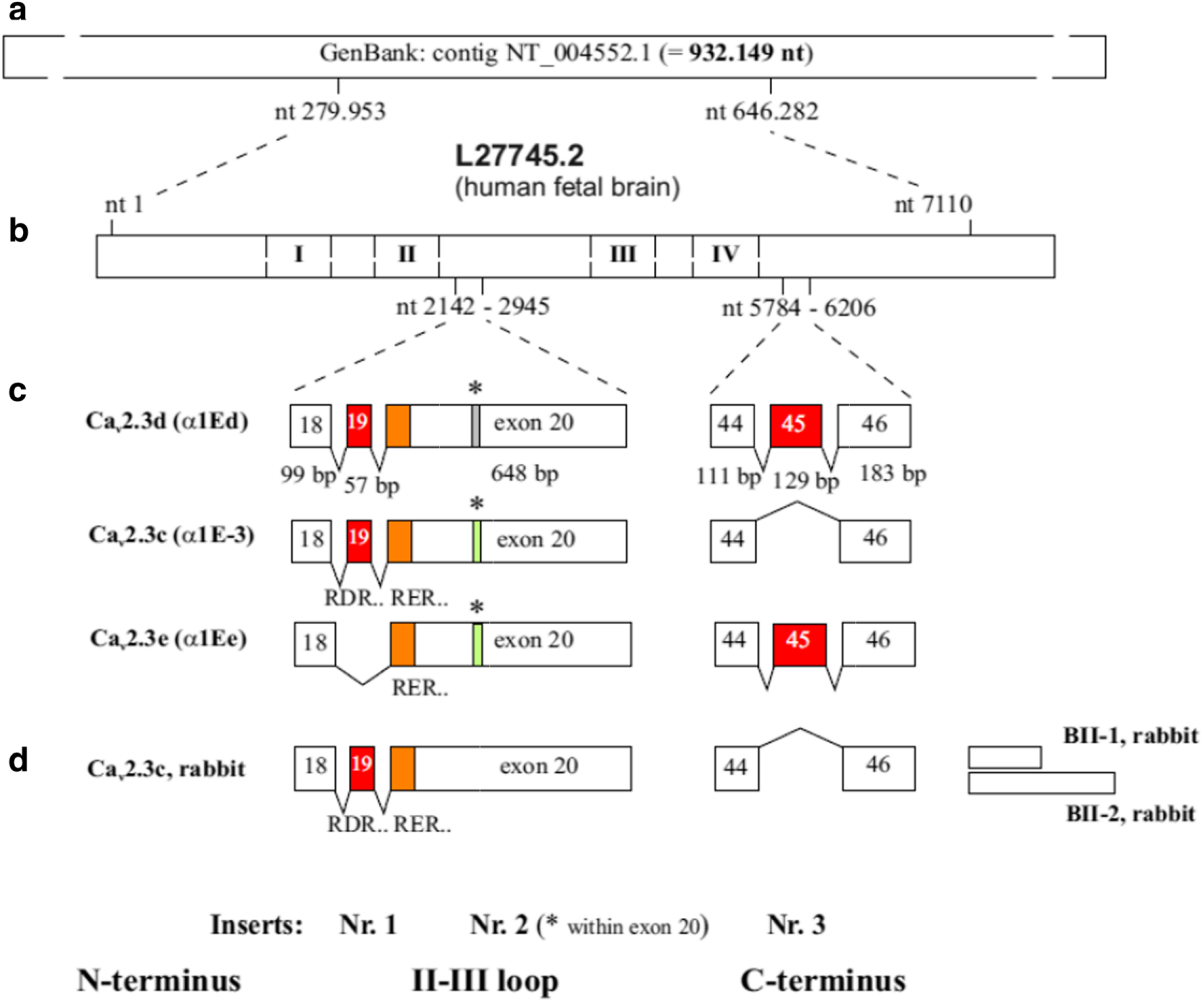
Splice variants of Cav2.3 calcium channels. In the carboxyterminal region, 2 splice variants were found, from which BII-2 had a 272-aas-long insertion in the carboxyterminus

In 1992, this novel R-type Ca^2+^ channel was initially detected from a rabbit brain, which was named the BII calcium channel [25]. Its primary sequence was deduced by molecular homology cloning and sequencing of cDNA. Its transcripts were found to be distributed predominantly in the cerebral cortex, hippocampus and corpus striatum. In the carboxyterminal region, 2 splice variants were found, from which BII-2 had a 272-aas-long insertion in the carboxyterminus (Fig. 2).

In 1993, a structural homolog of the new calcium channel was cloned from marine ray [12, 15, 43] and rat brain [37], which at that time was functionally expressed for the first time. Although the sequence of the rat rbE-II was structurally related to high voltage-activated Ca^2+^ channels, it was assumed to be a low-voltage-gated Ca^2+^ channel, because the rbE-II channel transiently activated at more negative membrane potentials, required a strong hyperpolarization to deinactivate and was highly sensitive to Ni^2+^ block. In situ hybridization showed that rbE-II messenger RNA was expressed in regions throughout the central nervous system [37]. Its predicted shorter N-terminal sequence was not confirmed by RT-PCR studies [34].

During the same time, a rapidly inactivating neuronal Ca^2+^ channel was identified, called doe-1 in ray (*Discopyge ommata*). Its expression showed that it was a high-voltage-activated Ca^2+^ channel that inactivated more rapidly than other known channel types like L- or P-type channels. It was insensitive towards dihydropyridine antagonists or the peptide toxin omega-Aga-IVa, respectively. This channel with novel functional properties was also sensitive towards micromolar Ni^2+^ concentrations as well as sensitive towards omega-conotoxin GVIA in a reversible manner [12]. At that time, a similar Ca^2+^ channel current was identified in rat cerebellar granule neurons [43], which was distinguished from T-type Ca^2+^ channels in dissociated neurons from native tissues [31].

In 1994, two human Cav2.3 sequences were published [33, 41] and both constructs were functionally expressed. In the same year, also the rabbit BII-2 variant and the mouse construct (Table 1) were identified. Additional and novel splice variants were amplified by RT-PCR [38] and full-length cloning [18, 21, 22], which showed that structural and functional details are important in different tissues. The functional implications have only partially been characterized [17, 19].

## Changes of Ca_v_2.3 transcript and expression levels in mouse models related to Parkinson disease

The neuronal Ca^2+^ sensor protein (NCS) was identified to be important for the viability and pathophysiology of dopaminergic (DA) midbrain neurons [11]. In a mouse model lacking NCS type 1 (NCS-1), several mitochondrial encoded proteins were reduced on the transcriptional level. Also, lower levels of Ca_v_2.3 were detected in substantia nigra (SN) neurons from NCS-1 KO mice [36], leading to a deeper analysis of the role of Ca_v_2.3 during the selective degeneration of DA midbrain neurons.

In an *in* vivo mouse model of Parkinson disease (injection of MPTP/probenecid), Ca_v_2.3 was identified as a mediator of SN dopaminergic neuron loss [5]. In adult SN dopaminergic neurons, it was shown that Ca_v_2.3 represents the most abundantly expressed voltage-gated Ca^2+^ channel subtype. It was linked with metabolic stress in these neurons or with their degeneration in Parkinson’s disease, which may occur by affecting Ca_v_-mediated Ca^2+^ oscillations and/or by changing Ca^2+^-dependent after hyperpolarizations (AHPs) in SN dopaminergic neurons [5].

## Tonic inhibition of Ca_v_2.3 by bioavailable divalent cations (Zn^2+^, Cu^2+^)

Ca_v_2.3/R-type Ca^2+^ channels are highly sensitive towards bioavailable divalent cations [23]. In the organotypic model of the isolated and superfused bovine retina, Ca_v_2.3/R-type Ca^2+^ channels have early been identified to be modulated by Zn^2+^ [35] and Cu^2+^ [24], Lüke et al., in press) changing the transretinal signalling. Zn^2+^ and Cu^2+^ effects can be analysed more specifically in heterologous expression systems, which have shown that e.g. Zn^2+^ can either increase or inhibit Ca^2+^ currents mediated by recombinant Ca_v_2.3 channels (Neumaier et al., unpublished results).

*In vivo*, Ca_v_2.3/R-type Ca^2+^ channels are thought to be under tight allosteric control by endogenous loosely bound trace metal cations (Zn^2+^ and Cu^2+^) that suppress channel gating via a high-affinity trace–metal-binding site. Unexpectedly, in wild-type mice the intracerebroventricular administration of histidine (1 mM) rather than Zn^2+^ itself in micromolar concentrations is beneficial during experimentally induced epilepsy [2]. In Ca_v_2.3-deficient mice, no beneficial effect of histidine is found and the experimentally induced seizures are less severe when Zn^2+^ in the presence of histidine is injected intracerebroventricularly [3].

As highly selective Cav2.3/R-type antagonists are still missing, the indirect modulation of Ca_v_2.3 by manipulation of bioavailable cation levels may provide an additional pathway for beneficial modulation of Ca_v_2.3/R-type Ca^2+^ channels.
